# Human endogenous retrovirus K (HERV-K) transcripts detection in babies exposed to HIV-1 during pregnancy

**DOI:** 10.1186/1742-4690-11-S1-P134

**Published:** 2014-01-07

**Authors:** Camila M Romano, Danielly Oliveira, Deborah Gerhardt, Luiz HS Nali, Giovana S Caleiro, Cristina F Nunes, Fernanda Granato, Fabiana B do Carmo, Regina CM Succi, Daisy M Machado

**Affiliations:** 1 Departamento de Moléstias Infecciosas e Parasitárias (LIMHC), Instituto de Medicina Tropical de São Paulo, Universidade de São Paulo, São Paulo, SP, Brazil; 2 Disciplina de Infectologia Pediátrica da Escola Paulista de Medicina, Universidade Federal de São Paulo, São Paulo, SP, Brazil

## 

Human endogenous retroviruses of the K family (HERV-K) are among the most recently integrated retroviruses in the human genome. HERV-K mRNA can be detected in normal tissues, but its expression is remarkably enhanced in HIV-1 infected patients due to HIV-Tat induction. Additionally, HERV-K activity stimulates both humoral and cellular responses, suggesting that HERV-specific immunity may contribute to the control of HIV-1 replication in adults. Recently, it was reported that vertically infected children has HERV specific T-cell response.

Since most children born from HIV infected mothers (65-85%) do not get infected despite the possible intra-utero contact with the virus, we evaluated the HERV-K activity in non-infected babies born from: HIV-1 positive mothers (HIV-exposed), and from non-HIV-1 mothers (non-exposed). Peripheral blood mononuclear cells (PBMC) were obtained from 11 babies between 1 to 11-months-old and also from their respective HIV-1 infected mothers. Exposed babies were not breastfed and had no HIV or other viral infection. HERV-K (HML-1-10) transcripts were screened by qRT-PCR, and the expression was evaluated through the absolute threshold-Ct normalized against B-Actin expression.

All babies presented some level of HERV-K expression that could not be differentiated between HIV-exposed and non-exposed groups. The expression was also similar according to the age and ethnic origin. Although all HIV-positive mothers have undetectable HIV-1 load, all but one expressed HERV-K. Thus, because all babies investigated presented HERV-K activity despite the HIV-1 exposure, it is possible that HERV activity in babies could works as a primal immunological mechanism to dealing with the exogenous infectious agents.

## Funding

This work was supported by FAPESP #2011/13612-9 and #2010/10619-0.

